# Cundurango—Answer to Correspondents

**Published:** 1871-09

**Authors:** 


### Editorial.

### Cundurango—Answer to Correspondents.

We have received numerous inquiries about this drug, which we desire to answer as satisfactorily as possible. This medicine can be obtained of Wm. Peabody, Druggist, Buffalo. It is worth one hundred dollars per pound, *only*. It has been qurte extensively tried in Buffalo, and six cases have been under our own observation, though we have not advised it. We have consented to prescribe it for the gratification of patients. After trying it about one week, and observing no effects, three of the patients discontinued its use. One other could not retain it on the stomach, or any other medicine or food. One of the remaining patients thought it relieved the pain, previously very intense, but a few day's trial dissipated the idea, the pain returned and the disease constantly increases while the medicine is still continued. The remaining patient continues the use ofthe drug, and *hopes* still to derive benefit from its continued use; no positive evidences of benefit are yet observable. So fabulous a story in medicine could hardly be expected to receive any credence in the profession, and we frankly confess to a disgust with the perpetrators of the statements furnished the daily press. We have steadfastly advised poor people to let it alone until the better off ones had tested its virtues. A Physician, backed by the Vice-President of the country, and a female savage of South America, can of course, make an impression upon the poor victims of malignant disease. It is not strange, with the statements furnished the country, that such patients and their friends should have intense desire to *try* the remedy. There is probably money enough to be made out of it to pay one Physician, well, for all his professional reputation and standing, and have enough left to fully reward the Vice-President for any outside influence he might have been able to furnish the enterprise We have designed to wait for the results of complete trial before expressing any opinions upon the subject, but too long delay in such a matter is criminal. We shall answer the inquiries made about this drug more fully after longer observation, and only desire to say, that we have seen no effects from the drug whatever, and believe it inert. Our professional associates express similar views, and we deem it but just to the profession and all honest seekers for the truth, thus early to make the expression.

We find the following editorial in the *Leavenworth Journal of Pharmacy*, which we re-publish for the entertainment ot our readers :

"WHAT'S IN A NAME?—High sounding titles; a six-in-hand turnout with gold mounted harness; specious pretentions—one and all are most wonderfully successful in the domain of quackery. Although "mere springs to catch woodcocks," it is astonishing how much of that kind of game is yearly trapped.

The magic power of ST.—1860—X, brought millions to the coffers of the genius who invented it. Some brilliant genius has devised a well laid scheme to enrich himself at the expense of suffering humanity by the introduction of an article for the cure of cancer. The name of the article is a taking one:—Cundurango sounds grandly when properly pronounced. It requires a peculiar twist of the tongue to give it full effect, and is all the more effective on that account. The term would be more euphonious if spelled with a *j* instead of a *d*—certainly it would be then more expressive of its object. Those who are to be conjured out of their dollars would have the satisfaction of knowing that it was a mere slight-of-hand pe:formance. Coming from a foreign country, introduced through high official channels, recommended by one of the leading physicians of the Capitol, and endorsed by so high a dignitary as the Vice-President of the United States, it would be strange indeed if it did not have a large sale. Unfortunately, however, four of the six persons upon whom its extraordinary virtues were tried, suddenly took their departure to a foreign clime, with the mere ghost of a chance of their return, and the other two are preparing to make the same journey, determined, however, to shout *cundurango!* with their last expiring breath.

Were the humbug not so transparent, we would cry shame on its perpetrators; but with the facts before us, we are inclined to pity them for their folly. In this connection we publish the following from our Arrapahoe poet, both as a compliment to the author and as a contribution to high art:

The morning sun was shining bright
As lone upon old Georgetown's hight,
A Bliss-ful doctor, clad in brown,
Desiring wealth and great renown,
Displayed aloft to wond'ring eyes
A shrub which bore this strange device,
Cundurango!

A maiden fair with palid cheek
With ardent haste his aid did seek
To stay the progress and the pain
Of carcinoma of the brain;
While still aloft the shrub he bore,
The answer came with windy roar,
Try Cundurango!

A matron old, with long unrest
From carcinoma of the breast,
This Bliss-ful doctor rushed to see,
And begg[illegible]d his aid on bended knee.
The magic shrub waved still on high,
And rushed through air the well-known cry,
Try Cundurango!

The evening sun went down in red—
The maid and matron both were dead;
And yet through all the realms around,
This worthless shrub, of mighty sound,
Will serve to fill the purse forlorn,
And cancer succumb—"in a horn"
To Cundurango."

